# Simvastatin Attenuates Doxorubicin-Induced Inflammation in Human Cardiomyocytes

**DOI:** 10.3390/biomedicines14051071

**Published:** 2026-05-08

**Authors:** Roberta Vitale, Rosaria Margherita Rispoli, Maria Carmela Di Marcantonio, Barbara Pala, Stefania Marzocco, Gabriella Mincione, Ada Popolo

**Affiliations:** 1 Department of Pharmacy, University of Salerno, 84084 Fisciano, Italy; rvitale@unisa.it (R.V.); rorispoli@unisa.it (R.M.R.); smarzocco@unisa.it (S.M.); 2 PhD Program in Drug Discovery and Development, University of Salerno, Via Giovanni Paolo II 132, 84084 Fisciano, Italy; 3 Department of Innovative Technologies in Medicine and Dentistry, University “G. d’Annunzio” Chieti-Pescara, 66100 Chieti, Italy; dimarcantonio@unich.it; 4 UOC Cardiologia Ospedale IDI-IRCCS, Via Monti di Creta 104, 00167 Roma, Italy; b.pala@idi.it; 5 PhD School of Applied Medical-Surgical Sciences, Tor Vergata University of Rome, Via Montpellier 1, 00133 Rome, Italy

**Keywords:** doxorubicin, simvastatin, cardiotoxicity, inflammation, inflammasome, connexin43

## Abstract

**Background/Objectives**: Clinical application of Doxorubicin (Doxo) is limited by cardiotoxicity, a process strongly associated with an interplay between oxidative stress and inflammatory signaling, particularly Nuclear Factor kappa-light-chain-enhancer of activated B cells (NF-κB) activation and Nucleotide oligomerization domain-like receptor family, pyrin domain containing 3 (NLRP3) inflammasome engagement. Identifying strategies capable of mitigating these interconnected pathways is of critical importance in cardio-oncology. Simvastatin (SIM) is a promising option since it modulates oxidative stress, inflammation, and cell death through its pleiotropic effects, so this study aimed to evaluate whether SIM attenuates Doxo-induced inflammatory responses. **Methods**: Human Cardiomyocyte (HCM) cells were pre-treated with SIM (10 µM) for 4 h and then co-exposed to SIM and Doxo (1 µM) for 20 h. Cytofluorimetric analysis was used to evaluate inducible nitric oxide synthase (iNOS), Connexin 43 (Cx43), and Cx43 phosphorylated at Serine 368 (p^S368^Cx43) levels. Real-time qPCR was performed to evaluate iNOS gene expression, while Nitric oxide (NO) release was evaluated by spectrophotometric analysis. Interleukin (IL)-1β, IL-18, IL-6, tumor necrosis factor alpha (TNF-α) production, and NLRP3 levels were evaluated by means of ELISA assay. Expression levels of inhibitor of nuclear factor kappa B alpha (IκB-α), Caspase-1, and Gasdermin D (GSDMD) were evaluated by Western Blot analysis. Nuclear translocation of NF-κB was evaluated by immunofluorescence assay. **Results**: In our experimental model, SIM significantly (*p* < 0.01) reduced Doxo-induced nitrite release, as well as iNOS gene expression (*p* < 0.05) and protein levels (*p* < 0.01). SIM also markedly attenuated Doxo-induced NF-κB signaling, pro-inflammatory cytokines production (TNF-α and IL-6, *p* < 0.01), and inflammosome-related responses (cleaved caspase-1, IL-1β, N-terminal domain of GSDMD), and NLRP3 expression *p* < 0.05). Additionally, SIM significantly attenuated the overexpression of Cx43 and its phosphorylated form (p^S368^Cx43), which are responsible for impairing intercellular communication and electrical coupling in cardiomyocytes and contribute to arrhythmias and conduction abnormalities characteristic of acute Doxo-induced cardiotoxicity. **Conclusions**: Overall, these findings demonstrate that SIM exerts a multifaceted cardioprotective effect against Doxo-induced injury, thereby targeting interconnected inflammatory and pro-arrhythmic pathways implicated in Doxo cardiotoxicity.

## 1. Introduction

Doxorubicin (Doxo) is a potent anthracycline chemotherapeutic agent extensively used in treating a broad spectrum of malignancies, including hematologic cancers and solid tumors [1]. Despite its efficacy, its clinical utility is substantially limited by cardiotoxicity, which can present as either acute or chronic forms [2]. Acute cardiotoxicity usually occurs within days of Doxo administration [3] and clinically manifests as transient arrhythmias and QT-interval prolongation. Occasionally, it can manifest as myopericarditis or pericardial involvement [4]. While these changes are usually mild and reversible, residual damage to cardiomyocytes may persist, leading to latent remodeling and potentially overt cardiomyopathy [2,5]. The mechanisms underlying acute cardiotoxicity are multifaceted and include oxidative stress, mitochondrial dysfunction, DNA damage (including that caused by topoisomerase IIβ), disturbances in calcium handling, and apoptotic cell death. However, growing evidence suggests that inflammation is a central and potentially modifiable contributor to cardiac injury [6]. Several studies suggest that inflammation is not merely a bystander in Doxo-induced cardiac injury, but a critical driver that is closely related to and interdependent with oxidative stress [7]. Indeed, innate immune signaling, involving pattern recognition receptors and downstream effectors, is activated in response to Doxo-induced damage. Endogenous molecules related to Doxo-induced mitochondrial damage are released as damage-associated molecular patterns (DAMPs) and are recognized via Toll-like receptor (TLR)-4, subsequently upregulating the Nuclear Factor kappa B (NF-κB) pathway [8]. Furthermore, Doxo activates the TLR2-Myeloid Differentiation Primary Response 88 (MyD88) complex, leading to the activation of NF-κB and the release of Tumor necrosis factor α (TNF-α), Interleukin-IL1β, IL-6, and IL-8, thereby contributing to the cardiac inflammatory process [9]. Sustained activation of these pathways amplifies inflammatory responses, promotes fibrotic remodeling, and contributes to progressive cardiac impairment [5]. Conversely, oxidative and nitrosative stress further reinforce inflammatory signaling. Elevated Reactive oxygen species (ROS), sarcoplasmic reticulum (ER) stress, and increased mitochondrial inducible Nitric oxide synthase (iNOS) levels, induced by Doxo administration, activate the NF-κB pathway, resulting in the production of pro-inflammatory cytokines, such as IL-6 and IL-1 [9]. These cytokines can impair mitochondrial function and reduce the activity of antioxidant enzymes, thereby increasing ROS production [10]. Furthermore, Doxo induces over-expression of iNOS, resulting in increased levels of nitric oxide (NO) that can react with superoxide (O_2_^−^) to form peroxynitrite (ONOO^−^). This highly reactive and damaging molecule can cause lipid peroxidation and DNA damage, as well as increasing oxidative/nitrosative stress in a destructive feedback loop. These effects further contribute to mitochondrial dysfunction, apoptosis, and ultimately cardiomyocyte death [11]. Given the interdependence of oxidative stress and inflammation in Doxo-induced cardiotoxicity (DIC), targeting both processes simultaneously could provide cardioprotective benefits. Based on their pleiotropic effects operating through multiple pathways implicated in the pathogenesis of chemotherapy-induced cardiotoxicity, Statins could be a valuable therapeutic strategy in the setting of DIC [12]. Furthermore, growing evidence indicates that the pleiotropic effects of Statins also include antiproliferative effects, inhibition of cell proliferation, and reduction of cancer progression [13,14], supporting their use as adjuvants in chemotherapy. Recently, our group showed that Simvastatin (Sim) significantly prevents Doxo-induced oxidative damage in a cellular model of Doxo-induced short-term cardiotoxicity [15]. Building upon these findings, the present study aimed to investigate whether SIM can also modulate Doxo-induced inflammatory and inflammasome activation under the same experimental conditions.

## 2. Materials and Methods

### 2.1. Cell Line and Treatment 

Human Cardiomyocyte (HCM) cell line was obtained from Celprogen (Huissen, The Netherlands; Benelux) and was maintained in 100 mm Corning dishes in the presence of Human Cardiomyocyte Cell Culture Complete Growth medium (M36044-15S, Celprogen) in a humidified incubator at 37 °C with 5% CO_2_. Cells were seeded at a density required for each experimental analysis and, after 24 h of adhesion, were pre-treated with Simvastatin (SIM 10 µM, #S6196 Sigma, Milan, Italy) for 4 h, followed by co-exposure to SIM and Doxorubicin (Doxo 1 µM, #S-5040420001 Sigma-Italy) for 20 h.

### 2.2. Intracellular Pro-Inflammatory Cytokines and NLRP3 Quantification

The enzyme-linked immunosorbent assay (ELISA) method was used to evaluate the expression of pro-inflammatory cytokines and of Nucleotide oligomerization domain-like receptor family, pyrin domain containing 3(NLRP3). After treatment, HCM cells (5 × 10^5^ cells/well into a 12-well plate) were lysed according to the protocol provided with the ELISA kits for IL-6 (E-EL-H6156), IL-18 (E-EL-H0253), IL-1β (E-EL-H0149), TNF-α (E-EL-H0109), and NLRP3 (E-EL-H2557). Briefly, freeze/thawing processes were performed in PBS containing protease inhibitors (E-EL-SR002, Elabscience, Houston, TX, USA). After centrifugation for 10 min at 1500× *g* at 4 °C, the resulting supernatant was collected for the assay. The levels of IL-1β, IL-6, IL-18, TNF-α, and NLRP3 were measured using a microplate spectrophotometer (Multiskan FC; Thermoscientific, Segrate (MI), Italy) equipped with a 450 nm filter. Total protein concentration (mg/mL) was estimated according to Bio-Rad Protein Assay (Bio-Rad, Milan, Italy), and ELISA results were normalized to protein content. Data are expressed as pg/mg or ng/mg of protein. 

### 2.3. Western Blotting Assay

HCM (5 × 10^5^ cells/well) were seeded into 12-well plates and treated as previously described. Proteins were extracted from the cells by freeze/thawing in RIPA buffer (1% Triton X, 0,25% Na-deoxycholate, 150 mM NaCl, 50 mM NaF, 1 mM EDTA, 0.2 mM Na_3_VO_4_, protease inhibitor, 50 mM Tris–HCl, pH 7.4). Protein content was estimated using the Bio-Rad Protein Assay (Bio-Rad, Milan, Italy) with bovine serum albumin as the standard. Then, 30 µg protein/lane were loaded onto an acrylamide gel and separated by SDS-PAGE in denaturing conditions. Blots were incubated overnight with primary antibody anti-IκB-α (sc-1643, Santa Cruz Biotechnologies, Dallas, TX, USA; diluted 1:250), anti-GSDMD (PA5-115330, Invitrogen, Segrate (MI), Italy; diluted 1:500) or anti-caspase-1 (sc-56036, Santa Cruz; diluted 1:250). Primary antibody anti-GAPDH (sc-137179, Santa Cruz; diluted 1:250) or anti-β-actin (E-AB-20031, Elabscience, Houston, TX, USA; diluted 1:2000) was used as a loading control. After incubation with the primary antibodies and washing in PBS/0.1% Tween, the appropriate secondary antibody, either anti-mouse (diluted 1:25,000) or anti-rabbit (diluted 1:5000), was added for 1 h at room temperature. Immunoreactive protein bands were detected using enhanced chemiluminescence reagents (ECL) in a LAS 4000 (GE Healthcare, Milan, Italy) system.

### 2.4. Immunofluorescence Analysis with Confocal Microscopy

For immunofluorescence analysis, HCM cells (5 × 10^4^ cells/well) were plated on coverslips in a 24-well plate and treated as previously described. After treatment, cells were fixed in 4% of formaldehyde, permeabilized with 0.1% Triton X-100, and blocked with 5% BSA (all from Sigma, Milan, Italy). Cells were then incubated overnight at 4 °C with a mouse monoclonal antibody against NF-κB p65 (sc-8008, Santa Cruz; diluted 1:250). FITC anti-mouse (1:250) was used for 1 h 30 min at room temperature (RT) in the dark. Dapi (1:3000) was used for counterstaining of nuclei. The coverslips were vertically scanned from the bottom by using a 40× Plan-Apochromat oil immersion objective. The images, which are shown as a single stack, and the scale bars were generated using Zeiss LSM 510 META Confocal Software (Release version 4.0 SP1, Carl Zeiss MicroImaging GmbH, Jena, Germany).

### 2.5. Flow Cytometry Analysis 

To assess the membrane levels of Connexin 43 (Cx43), HCM cells (1 × 10^4^ cells/well into 96-well plate) were treated as previously reported and then incubated with Fixing Buffer (PBS containing 1% BSA, 1% Formaldehyde) at 4 °C for 20 min. Thereafter, anti-Cx43 antibody (sc-9059, Santa Cruz; 1:250) was added along with the anti-rabbit FITC secondary antibody (1:250) for 30 min at 4 °C. Instead, to assess intracellular levels of Cx43 phosphorylated on Ser368 (p^S368^Cx43**)** and of inducible nitric oxide synthase (iNOS) after the incubation period at 4 °C for 20 min with Fixing buffer a permeabilization buffer (Fixing buffer containing 0.1% TritonX-100) was added and cells were incubated for further 30 min at 4 °C. Thereafter, anti- p^S368^Cx43 (CAT 48-3000, Invitrogen, Segrate (MI), Italy; 1:250) and anti-iNOS (sc-7271, Santa Cruz; 1:250), and anti-mouse FITC secondary antibody (1:250) were added at 4 °C for 30 min. The cells were then collected with Fixing buffer, and the fluorescence was evaluated using a fluorescence-activated cell sorting device (FACSscan; Beckton Dickinson, Milan, Italy) and processed using Cell Quest software (version number 5.2.1). The results are shown as the % of positive cells.

### 2.6. Measurement of Nitrite/Nitrate (NOx) Concentration

Nitrite (NO_2_^−^) levels, index of NO release, by Griess reaction [16]. Briefly, HCM cells (1 × 10^4^ cells/well into 96-well plate) were treated as previously described and then 100 µL of cell culture medium were mixed with 100 µL of Griess reagent (equal volumes of 1% (*w*:*v*) sulphanilamide in 5% (*v*:*v*) phosphoric acid and 0.1% (*w*:*v*) naphtylethylenediamine-hydrogen chloride) and incubated at room temperature for 10 min. The optical densities at 550 nm were then measured using a microplate spectrophotometer (Multiskan FC, Thermoscientific, Segrate (MI), Italy). Total NO_2_^−^ concentrations (µM) were calculated from the standard curve with sodium nitrate.

### 2.7. RNA Extraction and Real-Time qPCR of iNOS

Total RNA was extracted from cells treated as previously reported, using Trifast Reagent (EuroClone S.p.A., Pero, MI, Italy) in agreement with the manufacturer’s protocol. RNA concentration and purity were determined using the Nanodrop 1000 Spectrophotometer (Applied Biosystems, Thermo Fisher Scientific, Waltham, MA, USA), and complementary DNA (cDNA) was synthesized using the GoTaq^®^ 2-Step RT-qPCR System (Promega, Milan, Italy), following the manufacturer’s protocol. Quantitative real-time PCR (qRT-PCR) was performed using SYBR Green chemistry on a StepOne™ 2.0 Real-Time PCR System (Applied Biosystems, Carlsbad, CA, USA) and each reaction (10 μL) containing 1 μL template cDNA, 0.2 μL of primer mixture, and 5 μL of GoTaq^®^ 2-Step RT-qPCR System (Promega).

The thermal cycling conditions were performed as follows: initial denaturation at 95 °C for 10 min, followed by 40 cycles at 95 °C for 15 s and 60 °C for 1 min, and final elongation at 95 °C for 15 s. Relative gene expression levels were calculated using the comparative Ct method (2^−ΔΔCt^) and are presented as mean ± standard deviation (SD). Target gene expression, iNOS, was normalized by the ratio of the median value of the endogenous housekeeping, GAPDH, obtained in each treated cell vs. untreated cell. All experiments were performed in triplicate and repeated at least three times independently.

### 2.8. Statistical Analysis

Data are reported as the mean ± S.E.M. for at least three independent experiments, each performed in triplicate. The Shapiro-Wilk test was performed to verify data normality. Statistical analysis was performed by One-way analysis of variance (ANOVA) followed by Bonferroni’s multiple comparisons test, with the aid of the commercially available software GraphPad Prism 8 (GraphPad Software Inc., San Diego, CA, USA). Tukey’s multiple comparisons test was used for the qRT-PCR analysis. A *p*-value less than 0.05 was considered statistically significant.

## 3. Results

### 3.1. Effects of SIM Co-Treatment on Doxo-Induced NO Production and iNOS Expression

**Figure 1:**
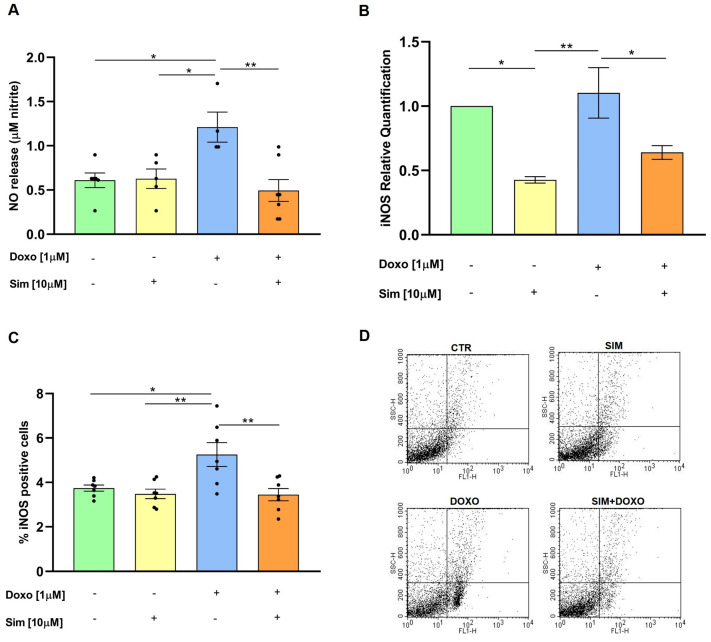
SIM co-treatment reduces Doxo-induced NO production and iNOS gene expression and protein levels. HCM were treated with SIM (10 µM) for 4 h and co-treated with Doxo (1 µM) for 20 h. A spectrophotometric assay was performed to evaluate NO release. Values are expressed as mean ± SEM of µM nitrite release (**A**). Real-time qPCR was used to evaluate iNOS gene expression. Data were calculated using the 2^−ΔΔCt^ method, normalized to GAPDH cDNA levels, and then expressed relative to control (calibrator sample, defined as 1.00) (**B**). Data are expressed as means ± SD and were analyzed by analysis of variance (ANOVA) followed by Tukey’s multiple comparisons test. Cytofluorimetric analysis was performed to evaluate iNOS levels. Values are expressed as mean ± SEM of % of iNOS positive cells (**C**). Statistical analysis was performed using One-Way ANOVA followed by the Bonferroni multiple comparisons test. Representative histograms for the flow cytometry analysis are reported in Panel (**D**). * *p* < 0.05, ** *p* < 0.01.

In the heart, Doxo promotes nitric oxide (NO) production by increasing the expression of iNOS protein and mRNA levels. This subsequently leads to the formation of reactive nitrogen species, contributing to nitrosative stress and amplification of the inflammatory process [11,17]. We therefore analyzed NO production, as well as iNOS gene expression and protein levels, to determine whether SIM co-treatment could mitigate Doxo-induced effects in our experimental model. Spectrophotometric analysis showed that SIM co-treatment significantly (*p* < 0.01) reduced nitrite release in the cell medium, an indirect parameter of NO production, induced by Doxo (Figure 1A). Moreover, data from real-time qPCR and cytofluorimetric analysis revealed that SIM co-treatment significantly decreased the overexpression of both the iNOS gene (*p* < 0.05, Figure 1B) and protein levels (*p* < 0.01, Figure 1C) induced by Doxo.

### 3.2. Effects of SIM Co-Treatment on Doxo-Induced NF-κB Signaling and Pro-Inflammatory Cytokines Production

**Figure 2:**
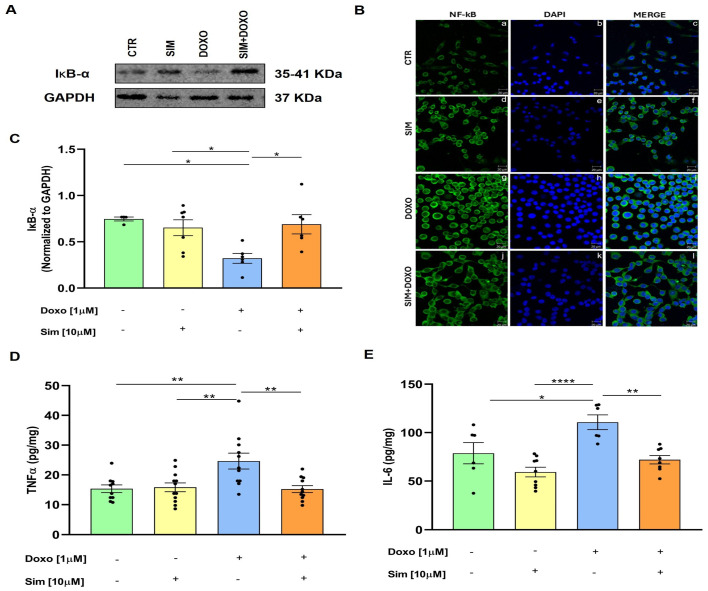
SIM co-treatment attenuates Doxo-induced NF-κB pathway activation and pro-inflammatory cytokine production. HCM were treated with SIM (10 µM) for 4 h and co-treated with Doxo (1 µM) for 20 h. IκB-α expression was detected by Western blot analysis, and GAPDH expression was used as the loading control (**A**,**C**). Values are expressed as mean ± SEM of optical density in at least three independent experiments. Cells were stained with NF-κB (green) (panels **a**,**d**,**g**,**j**) and nucleus with DAPI (blue) (panels **b**,**e**,**h**,**k**), and were determined by immunofluorescence analysis (merge in panels **c**,**f**,**i**,**l**). Scale bar, 20 µm (**B**). Intracellular TNF-α and IL-6 levels were evaluated by means of ELISA assay (**D**,**E**). Values are expressed as mean ± SEM of pg/mg of TNF-α and IL-6 produced. Statistical analysis was performed using One-Way ANOVA followed by the Bonferroni multiple comparisons test. * *p* < 0.05, ** *p* < 0.01, **** *p* < 0.001.

Oxidative stress induced by Doxo triggers an inflammatory response characterized by activation of the NF-κB pathway and subsequent release of pro-inflammatory cytokines, including TNF-α and IL-6 [18,19,20]. Under resting conditions, the NF-κB p65 subunit binds to its inhibitory counterpart, IκBα, and other IκB proteins to form an inactive cytoplasmic complex. Upon activation, IκBα is phosphorylated, leading to the activation and translocation of NF-κB into the nucleus, where it regulates the expression of inflammatory genes [21]. To evaluate the effects of SIM co-treatment on Doxo-induced activation of the NF-κB pathway, Western blot analysis was performed, and our data showed that Doxo treatment significantly reduced (*p* < 0.05 vs. untreated cells) IκBα expression, while SIM co-treatment significantly (*p* < 0.05) reverted Doxo-induced effects (Figure 2C). Consistent with these findings, immunofluorescence analysis showed that SIM co-treatment mitigated Doxo-induced NF-κB nuclear translocation (Figure 2B, Panels i and l). Furthermore, an ELISA assay performed on cellular lysates showed that SIM co-treatment significantly reduced the overproduction of TNF-α (*p* < 0.01) and IL-6 (*p* < 0.01) induced by Doxo (Figure 2D,E).

### 3.3. Effects of SIM Co-Treatment on Doxo-Induced Inflammasome-Related Responses

**Figure 3:**
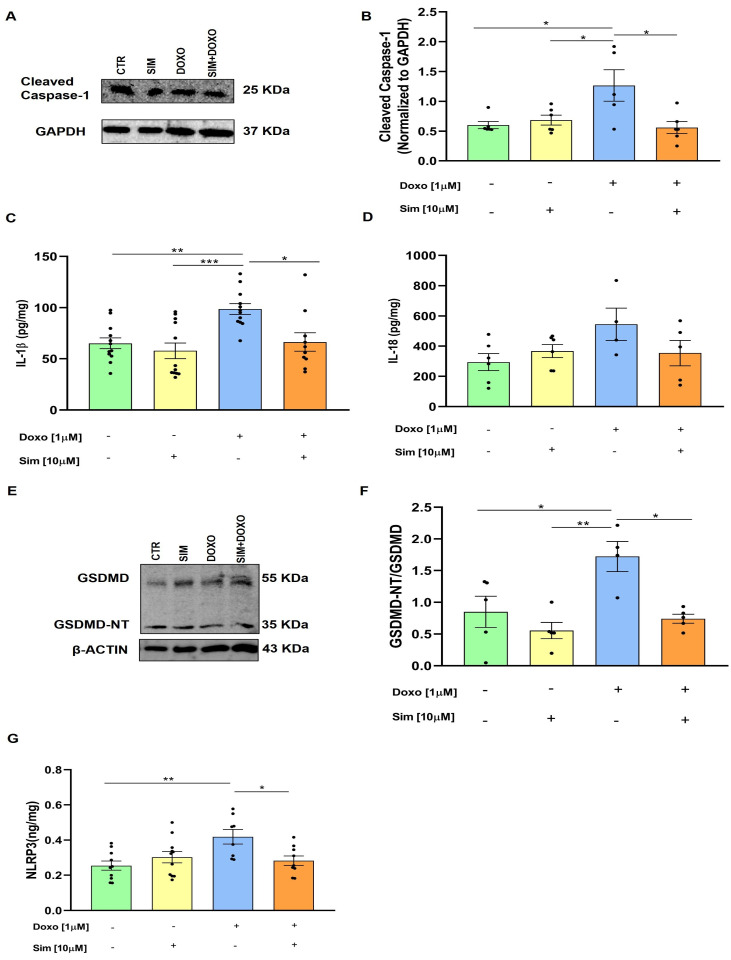
SIM co-treatment attenuates Doxo-induced inflammasome-related responses. HCM were treated with SIM (10 µM) for 4 h and co-treated with Doxo (1 µM) for 20 h. Cleaved Caspase-1 expression was detected by Western blot analysis, and GAPDH expression was used as the loading control. Values are expressed as mean ± SEM of optical density in at least three independent experiments (**A**,**B**). Intracellular IL-1β and IL-18 levels were evaluated by means of an ELISA assay. Values are expressed as mean ± SEM of pg/mg of IL-1β and IL-18 production (**C**,**D**). GSDMD and GSDMD-NT expression was detected by Western blot analysis, and β-actin expression was used as the loading control. Values are expressed as mean ± SEM of optical density in at least three independent experiments (**E**,**F**). Intracellular levels of NLRP3 were evaluated by means of an ELISA assay. Values are expressed as mean ± SEM of ng/mg of NLRP3 produced in at least three independent experiments (**G**). Statistical analysis was performed using One-Way ANOVA followed by the Bonferroni multiple comparisons test. * *p* < 0.05, ** *p* < 0.01, and *** *p* < 0.005.

Consistent findings across multiple studies support an association between DIC and the activation of NLRP3 inflammasomes. This leads to the activation of caspase-1, which promotes the maturation of pro-IL-1β and pro-IL-18 as well as the cleavage of Gasdemin-D [22]. Western blot analysis showed that SIM co-treatment significantly (*p* < 0.05) reduced cleaved caspase-1 expression, which was significantly (*p* < 0.05 versus untreated cells) increased in Doxo-treated cells (Figure 3A,B). Data from the ELISA assay, conducted to assess the intracellular concentration of IL-1β and IL-18 in our experimental model, showed that SIM co-treatment significantly reduced the overproduction of IL-1β (*p* < 0.05) induced by Doxo (Figure 3C). Regarding IL-18, we observed an increase in Doxo-treated cells and a decrease in SIM co-treated cells, even if no statistically significant differences were observed (Figure 3D). Furthermore, N-terminal GSDMD (GSDMD-NT) expression was evaluated by Western blot analysis, and our results showed that SIM co-treatment significantly (*p* < 0.05) reduced its overexpression induced by Doxo (Figure 3E,F). Finally, ELISA assay results showed that SIM co-treatment significantly reduced (*p* < 0.05) Doxo-induced NLRP3 expression (Figure 3G).

### 3.4. Effects of SIM Co-Treatment on Cx43 Levels

**Figure 4:**
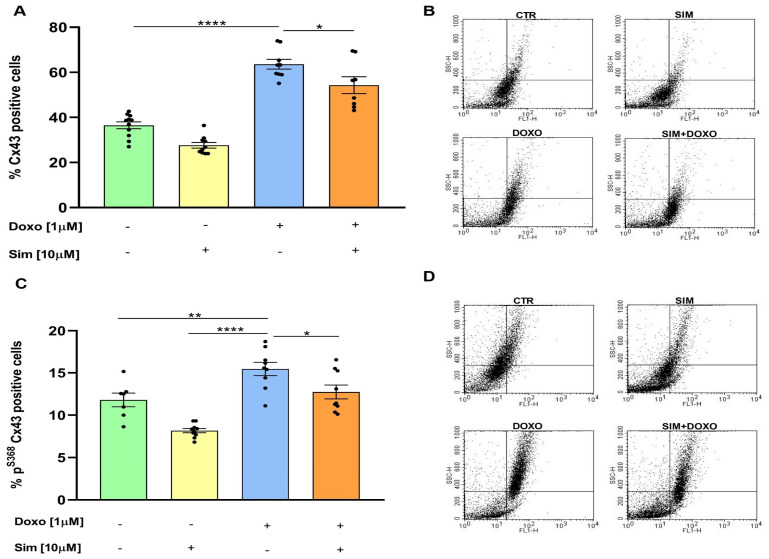
SIM co-treatment attenuates Doxo-induced Cx43 and p^S368^Cx43 overexpression. HCM were treated with SIM (10 µM) for 4 h and co-treated with Doxo (1 µM) for 20 h. To evaluate Cx43 and p^S368^Cx43 levels, flow cytometry analysis was used. The results are reported as the mean ± SEM of the percentage Cx43 and p^S368^Cx43 positive cells (**A**,**C**). Statistical analysis was performed using one-way ANOVA followed by the Bonferroni multiple comparisons test. Representative histograms for the flow cytometry analysis are reported in Panels (**B**,**D**). * *p* < 0.05, ** *p* < 0.01, and **** *p* < 0.001.

The cytokine hypothesis, formulated by Bozkurt in 2000, proposed that the overproduction of pro-inflammatory cytokines in the heart is associated with heart failure progression, initially characterised by cardiac arrhythmias [23]. As electrical coupling and intercellular communication in cardiac cells are regulated by Cx43-formed gap junctions, we used cytofluorimetric analysis to evaluate Cx43 levels in our experimental model. Our results showed that SIM co-treatment significantly (*p* < 0.05) reduced Doxo-induced Cx43 overexpression (*p* < 0.001 versus untreated cells) (Figure 4A). We also evaluated the levels of Cx43 phosphorylated on Ser368 (p^S368^Cx43), which is known to regulate the permeability of gap junctions formed by Cx43 [24]. As shown in Figure 4C, SIM co-treatment significantly (*p* < 0.05) attenuated the Doxo-induced increase in p^S368^Cx43 levels (*p* < 0.001 versus untreated cells).

## 4. Discussion

Cardiotoxicity is one of the most severe side effects of DOXO, significantly impacting the morbidity and mortality of cancer survivors. Currently, aside from Dexrazoxane, whose benefits remain controversial [25], there are no specific therapies for cardiotoxicity. Therefore, identifying pharmacological approaches capable of interfering with the early molecular events underlying Doxo-induced cardiac injury is a key translational objective [26]. In recent years, research has focused on the molecular mechanisms involved in the early stages of Doxo-mediated cardiotoxicity, since damage to cardiomyocytes during this phase leads to cardiac dysfunction that manifests clinically years after drug discontinuation [27]. There is growing evidence that Doxo-induced cardiotoxicity is closely associated with nitrosative and oxidative stress, which exacerbate inflammatory signaling and ultimately contribute to cardiac dysfunction. In a previous study, we demonstrated that SIM can prevent oxidative stress and subsequent apoptosis in Doxo-exposed cardiomyocytes [15]. Given statins’ numerous pleiotropic effects, including anti-inflammatory activity, this study aimed to evaluate whether SIM could also reduce Doxo-induced inflammation in a short-term cellular model. It is widely accepted that Doxo can upregulate nitric oxide (NO) synthesis in cardiac cells by increasing NOS expression [28]. Increased NO bioavailability promotes the formation of reactive oxygen species and amplifies nitrosative stress, thereby exacerbating inflammatory responses and contributing to contractile dysfunction and cardiomyocyte death [10]. Consistent with previous reports, we observed an increased nitrite accumulation (a stable NO metabolite) in the culture medium of Doxo-treated cells. Importantly, our data showed that SIM co-treatment significantly attenuates Doxo-induced NO production, suggesting a protective role for SIM against nitrosative stress. The reduction in NO levels observed in SIM co-treated cells is mechanistically supported by the significant downregulation of Doxo-induced iNOS overexpression at both mRNA and protein levels. As iNOS-derived NO is a major contributor to sustained inflammatory signaling under pathological conditions, the ability of SIM to suppress iNOS overexpression is a critical mechanism by which it mitigates Doxo-induced cellular stress. These findings are consistent with previous studies that have implicated iNOS inhibition as a key strategy for reducing inflammatory and nitrosative damage in cardiovascular models [29,30]. Oxidative and nitrosative stress induced by Doxo are known to activate the NF-κB signaling pathway, which plays a pivotal role in orchestrating inflammatory responses in the heart [31]. Under basal conditions, NF-κB is retained in the cytoplasm through its interaction with inhibitory IκB proteins. Upon activation, the phosphorylation and subsequent degradation of IκBα permit the nuclear translocation of NF-κB and the transcription of pro-inflammatory cytokines, including TNF-α and IL-6 [21]. In line with this paradigm, in our experimental model, Doxo-treatment resulted in a marked reduction of cytoplasmic IκB levels and enhanced NF-κB p65 nuclear localization, indicating pathway activation. Interestingly, SIM co-treatment restored IκBα levels and markedly reduced NF-κB nuclear translocation, suggesting that SIM effectively suppresses Doxo-induced NF-κB activation, as previously reported for other statins [32]. The functional relevance of NF-κB inhibition by SIM is further supported by the pronounced reduction in TNF-α and IL-6 overproduction, both of which are key mediators of cardiac inflammation and remodelling. These results highlight the capacity of SIM to blunt the downstream inflammatory signaling triggered by NF-κB activation, thereby limiting the inflammatory burden associated with Doxo exposure [33]. Emerging evidence suggests that Doxo-induced cardiotoxicity involves activation of the NLRP3 inflammasome, leading to caspase-1 activation, cytokine maturation, and pyroptotic cell death. Consistent with this paradigm, our data demonstrate that Doxo increases IL-1β and IL-18 production, as well as the expression of NLRP3, cleaved caspase-1, and the N-terminal fragment of Gasdermin D (GSDMD-NT), indicative of inflammasome activation and pyroptosis [34]. Importantly, SIM co-treatment significantly attenuated these effects, reducing caspase-1 activation, IL-1β overproduction, and NLRP3 expression. Although IL-18 levels exhibited a decreasing trend without reaching statistical significance, the overall suppression of inflammasome components suggests that SIM interferes with both the priming and activation phases of NLRP3 signaling. Furthermore, the observed reduction in GSDMD-NT expression further supports the notion that SIM limits inflammasome-mediated pyroptotic cell death, thereby preserving cardiomyocyte viability [35]. Elevated levels of pro-inflammatory cytokines have been shown to alter Cx43 expression [36,37]. Cx43 is the main gap junction protein in cardiac cells and is involved in synchronizing rhythmic contractions and maintaining cardiac homeostasis [38]. Phosphorylation of Cx43, particularly at serine 368, significantly impacts the behavior of gap junctions by reducing the open probability of Cx43 channels and limiting direct electrical and metabolic spread between cells. While increased expression of Cx43 and of its phosphorylated form is part of a compensatory response to maintain cellular function, the so-called “Good Samaritan effect”, prolonged Cx43 phosphorylation disrupts intercellular communication and predisposes the myocardium to transient arrhythmias and conduction abnormalities that clinically characterize Doxo-induced acute cardiotoxicity [39,40]. Here, we demonstrate that SIM co-treatment reduces the overexpression of both Cx43 and p^S368^Cx43 induced by Doxo, suggesting a restoration of gap junction homeostasis. These findings align with prior evidence indicating that SIM modulates Cx43 and p^S368^Cx43 in cardiomyocytes [38], thereby exerting anti-arrhythmic effects independently of its cholesterol-lowering activity. Indeed, it has been shown that the inhibitory effect of statins on Cx43 is associated with the suppression of pro-inflammatory cytokines [41], especially TNF-α, IL-1β, and IL-6 [42].

## 5. Conclusions

Overall, our study provides compelling evidence that SIM exerts a multifaceted cardioprotective effect against Doxo-induced cardiotoxicity. By simultaneously suppressing iNOS-dependent NO overproduction, inhibiting NF-κB-driven inflammatory signaling, restraining NLRP3 inflammasome activation, and normalizing Cx43 expression and phosphorylation, SIM targets key pathological processes that collectively contribute to Doxo-mediated cardiac dysfunction. This integrated mechanism of action is particularly relevant given the complex and interconnected nature of cardiotoxic signaling pathways. However, this study was conducted in a short-term in vitro model and did not directly assess functional contractility, electrophysiological properties, or long-term remodeling. Furthermore, it failed to consider the physiological complexity of the heart (e.g., interaction with fibroblasts or the extracellular matrix). Therefore, future studies are needed to further elucidate the upstream molecular targets of SIM and to validate these protective effects in in vivo models, thereby strengthening its potential translational relevance. Despite these limitations, our data provide mechanistic evidence supporting Simvastatin as a potential adjunctive strategy to mitigate Doxo-induced inflammatory cardiotoxicity.

## Data Availability

The raw data supporting the conclusions of this article will be made available by the authors on request.

## Abbreviations

The following abbreviations are used in this manuscript:

HCM	Human Cardiomyocytes
DOXO	Doxorubicin
SIM	Simvastatin
IL-6	Interleukin 6
IL-18	Interleukin 18
IL-1β	Interleukin 1 beta
TNF-α	Tumor necrosis factor alpha
IκB-α	Inhibitor of nuclear factor kappa B alpha
NF-κB	Nuclear Factor kappa-light-chain-enhancer of activated B cells
NLRP3	Nucleotide oligomerization domain-like receptor family, pyrin domain containing 3
GSDMD	Gasdermin D
NT-GSDMD	N-terminal domain of Gasdermin D
TLR-4	Tool-like receptor 4
TLR-2	Tool-like receptor 2
MyD88	Myeloid Differentiation Primary Response 88
Cx43	Connexin 43
p^S368^Cx43	Connexin43 phosphorylated at Serine 368
iNOS	Inducible nitric oxide synthase
NO	Nitric oxide

## References

[1] Jones I.C., Dass C.R. (2022). Doxorubicin-induced cardiotoxicity: Causative factors and possible interventions. J. Pharm. Pharmacol..

[2] Fabiani I., Chianca M., Cipolla C.M., Cardinale D.M. (2025). Anthracycline-induced cardiomyopathy: Risk prediction, prevention and treatment. Nat. Rev. Cardiol..

[3] Abushouk A.I., Salem A.M.A., Saad A., Afifi A.M., Afify A.Y., Afify H., Salem H.S.E., Ghanem E., Abdel-Daim M.M. (2019). Mesenchymal Stem Cell Therapy for Doxorubicin-Induced Cardiomyopathy: Potential Mechanisms, Governing Factors, and Implications of the Heart Stem Cell Debate. Front. Pharmacol..

[4] Tamakuwala K., Panchani N., Gupta A., Rawal J. (2015). Doxorubicin induced reversible cardiomyopathy: A Case report. J. Integr. Health Sci..

[5] Bosman M., Krüger D., Van Assche C., Boen H., Neutel C., Favere K., Franssen C., Martinet W., Roth L., De Meyer G.R.Y. (2023). Doxorubicin-induced cardiovascular toxicity: A longitudinal evaluation of functional and molecular markers. Cardiovasc. Res..

[6] Zhang Z., Du T., Wu N., Yang S., Wang J., Peng J., Jia Z., Dai J., Du X., Feng M. (2024). Sulfiredoxin 1 ameliorates doxorubicin-induced cardiotoxicity by suppressing oxidative stress and inflammation via the Sirt1/NLRP3 pathway. Int. Immunopharmacol..

[7] Negm A., Mersal E.A., Dawood A.F., Abd El-Azim A.O., Hasan O., Alaqidi R., Alotaibi A., Alshahrani M., Alheraiz A., Shawky T.M. (2025). Multifaceted Cardioprotective Potential of Reduced Glutathione Against Doxorubicin-Induced Cardiotoxicity via Modulating Inflammation–Oxidative Stress Axis. Int. J. Mol. Sci..

[8] Bhagat A., Shrestha P., Kleinerman E.S. (2022). The Innate Immune System in Cardiovascular Diseases and Its Role in Doxorubicin-Induced Cardiotoxicity. Int. J. Mol. Sci..

[9] Arrigoni R., Jirillo E., Caiati C. (2025). Pathophysiology of Doxorubicin-Mediated Cardiotoxicity. Toxics.

[10] Vitale R., Marzocco S., Popolo A. (2024). Role of Oxidative Stress and Inflammation in Doxorubicin Induced Cardiotoxicity: A Brief Account. Int. J. Mol. Sci..

[11] Bagchi A.K., Malik A., Akolkar G., Jassal D.S., Singal P.K. (2021). Endoplasmic Reticulum Stress Promotes iNOS/NO and Influences Inflammation in the Development of Doxorubicin-Induced Cardiomyopathy. Antioxidants.

[12] Arendt N., Kopsida M., Lennernäs H., Sjöblom M., Heindryckx M.F. (2025). Statin-mediated protection in chemotherapy-induced intestinal and cardiac toxicity: Current perspectives. Eur. J. Pharmacol..

[13] Vitale R., Marzocco S., Popolo A. (2025). Simvastatin Enhances the Cytotoxic Effects of Doxorubicin in a Mammary Adenocarcinoma Cell Model by Involving Connexin 43. J. Biochem. Mol. Toxicol..

[14] Mauriello A., Correra A., Maratea A.C., Fonderico C., Amata A., Cetoretta V., Russo V., D’Andrea A. (2025). Protective Role of Lipid-Lowering Drugs in Breast Cancer: Effects on Cancer Incidence and Cardiotoxicity. Life.

[15] Vitale R., Mazzone M., Di Marcantonio M.C., Marzocco S., Mincione G., Popolo A. (2025). Cardioprotective Effects of Simvastatin in Doxorubicin-Induced Acute Cardiomyocyte Injury. Int. J. Mol. Sci..

[16] Pecoraro M., Pala B., Di Marcantonio M.C., Muraro R., Marzocco S., Pinto A., Mincione G., Popolo A. (2020). Doxorubicin-induced oxidative and nitrosative stress: Mitochondrial connexin 43 is at the crossroads. Int. J. Mol. Med..

[17] Henninger C., Fritz G. (2017). Statins in anthracycline-induced cardiotoxicity: Rac and Rho, and the heartbreakers. Cell. Death Dis..

[18] Quagliariello V., Canale M.L., Bisceglia I., Iovine M., Paccone A., Maurea C., Scherillo M., Merola A., Giordano V., Palma G. (2024). Sodium-glucose cotransporter 2 inhibitor dapagliflozin prevents ejection fraction reduction, reduces myocardial and renal NF-κB expression and systemic pro-inflammatory biomarkers in models of short-term doxorubicin cardiotoxicity. Front. Cardiovasc. Med..

[19] Shi S., Chen Y., Luo Z., Nie G., Dai Y. (2023). Role of oxidative stress and inflammation-related signaling pathways in doxorubicin-induced cardiomyopathy. Cell Commun. Signal..

[20] TamehriZadeh S.S., Khalaji M., Tajdari M., Mavaddat H., Szmit S., Lashgari N.A., Roudsari N.M., Abbasi-Kashkoli H., Banach M., Abdolghaffari A.H. (2025). Statins: Novel Approaches for the Management of Doxorubicin-Induced Cardiotoxicity—A Literature Review. Cardiovasc. Toxicol..

[21] El-Agamy D.S., El-Harbi K.M., Khoshhal S., Ahmed N., Elkablawy M.A., Shaaban A.A., Abo-Haded H.M. (2018). Pristimerin protects against doxorubicin-induced cardiotoxicity and fibrosis through modulation of Nrf2 and MAPK/NF-kB signaling pathways. Cancer Manag. Res..

[22] Zhao X.P., Duan L., Zhao Q.R., Lv X., Tian N.Y., Yang S.L., Dong K. (2025). NLRP3 inflammasome as a therapeutic target in doxorubicin-induced cardiotoxicity: Role of phytochemicals. Front. Pharmacol..

[23] Bozkurt B. (2000). Activation of cytokines as a mechanism of disease progression in heart failure. Ann. Rheum. Dis..

[24] Zhang X.M., Liu Y.L., Cai Y., Hao Y., Kang S. (2023). LRP6-mediated phosphorylation of connexin43 in myocardial infarction. iScience.

[25] Zheng H., Zhan H. (2025). Dexrazoxane makes doxorubicin-induced heart failure a rare event in sarcoma patients receiving high cumulative doses. Cardio-Oncology.

[26] Timm K.N., Perera C., Ball V., Henry J.A., Miller J.J., Kerr M., West J.A., Sharma E., Broxholme J., Logan A. (2020). Early detection of doxorubicin-induced cardiotoxicity in rats by its cardiac metabolic signature assessed with hyperpolarized MRI. Commun. Biol..

[27] Cardinale D., Iacopo F., Cipolla C.M. (2020). Cardiotoxicity of Anthracyclines. Front. Cardiovasc. Med..

[28] Aldieri E., Bergandi L., Riganti C., Costamagna C., Bosia A., Ghigo D. (2002). Doxorubicin induces an increase of nitric oxide synthesis in rat cardiac cells that is inhibited by iron supplementation. Toxicol. Appl. Pharmacol..

[29] Wang Z.Q., Chen M.T., Zhang R., Zhang Y., Li W., Li Y.G. (2016). Docosahexaenoic Acid Attenuates Doxorubicin-induced Cytotoxicity and Inflammation by Suppressing NF-κB/iNOS/NO Signaling Pathway Activation in H9C2 Cardiac Cells. J. Cardiovasc. Pharmacol..

[30] Shabaan D.A., Mostafa N., El-Desoky M.M., Arafat E.A. (2023). Coenzyme Q10 protects against doxorubicin-induced cardiomyopathy via antioxidant and anti-apoptotic pathway. Tissue Barriers.

[31] Nsairat H., Lafi Z., Abualsoud B.M., Al-Najjar B.O., Al-Samydai A., Oriquat G.A., Alshaer W., Alqader Ibrahim A., Dellinger A.L. (2025). Vitamin C as a Cardioprotective Agent Against Doxorubicin-Induced Cardiotoxicity. J. Am. Heart Assoc..

[32] Wei C.Y., Huang K.C., Chou Y.H., Hsieh P.F., Lin K.H., Lin W.W. (2006). The role of Rho-associated kinase in differential regulation by statins of interleukin-1β- and lipopolysaccharide-mediated nuclear factor ĸB activation and inducible nitric-oxide synthase gene expression in vascular smooth muscle cells. Mol. Pharmacol..

[33] Ito-Hagiwara K., Hagiwara J., Endo Y., Becker L.B., Hayashida K. (2025). Cardioprotective strategies against doxorubicin-induced cardiotoxicity: A review from standard therapies to emerging mitochondrial transplantation. Biomed. Pharmacother..

[34] Ye B., Shi X., Xu J., Dai S., Xu J., Fan X., Han B., Han J. (2022). Gasdermin D mediates doxorubicin-induced cardiomyocyte pyroptosis and cardiotoxicity via directly binding to doxorubicin and changes in mitochondrial damage. Transl. Res..

[35] Yu S.Y., Tang L., Zhao G.J., Zhou S.H. (2017). Statin protects the heart against ischemia-reperfusion injury via inhibition of the NLRP3 inflammasome. Int. J. Cardiol..

[36] Barreto B.C., das Neves M.V.G., Cardoso C.M.A., Meira C.S., Daltro P.S., Figueira C.P., Santos G.C., Silva D.N., Távora F., Neto J.D.S. (2024). The effects of inflammation on connexin 43 in chronic Chagas disease cardiomyopathy. Front. Immunol..

[37] Pecoraro M., Del Pizzo M., Marzocco S., Sorrentino R., Ciccarelli M., Iaccarino G., Pinto A., Popolo A. (2016). Inflammatory mediators in a short-time mouse model of doxorubicin-induced cardiotoxicity. Toxicol. Appl. Pharmacol..

[38] Rodríguez-Sinovas A., Sánchez J.A., Valls-Lacalle L., Consegal M., Ferreira-González I. (2021). Connexins in the Heart: Regulation, Function and Involvement in Cardiac Disease. Int. J. Mol. Sci..

[39] Yasui K., Kada K., Hojo M., Lee J.K., Kamiya K., Toyama J., Opthof T., Kodama I. (2000). Cell-to-cell interaction prevents cell death in cultured neonatal rat ventricular myocytes. Cardiovasc. Res..

[40] Blanc M., Bruce-Keller A.J., Mattson M.P. (1998). Astrocytic gap junctional communication decreases neuronal vulnerability to oxidative stress-induced disruption of Ca^2+^ homeostasis and cell death. J. Neurochem..

[41] Lin Y.C., Chiang C.H., Chang L.T., Sun C.K., Leu S., Shao P.L., Hsieh M.C., Tsai T.H., Chua S., Chung S.Y. (2013). Simvastatin attenuates the additive effects of TNF-α and IL-18 on the connexin 43 up-regulation and over-proliferation of cultured aortic smooth muscle cells. Cytokine.

[42] Qu L., Jiang J., Kong W. (2014). Pharmacological Effects of Statins Related to Gap Junction Modulation. Pharmacol. Pharm..

